# Apathy in Parkinson’s Disease: Clinical Patterns and Neurobiological Basis

**DOI:** 10.3390/cells12121599

**Published:** 2023-06-10

**Authors:** Matthieu Béreau, Vincent Van Waes, Mathieu Servant, Eloi Magnin, Laurent Tatu, Mathieu Anheim

**Affiliations:** 1 Département de Neurologie, CHU de Besançon, 25000 Besançon, France; 2 Université de Franche-Comté, LINC Laboratoire de Recherches Intégratives en Neurosciences et Psychologie Cognitive, 25000 Besançon, France; 3 Laboratoire d’Anatomie, Université de Franche-Comté, 25000 Besançon, France; 4 Département de Neurologie, CHU de Strasbourg, 67200 Strasbourg, France; 5 Fédération de Médecine Translationnelle de Strasbourg (FMTS), Université de Strasbourg, 67000 Strasbourg, France; 6 Institut de génétique Et de Biologie Moléculaire Et Cellulaire (IGBMC), INSERM-U964, CNRS-UMR7104, Université de Strasbourg, 67400 Illkirch-Graffenstaden, France

**Keywords:** apathy, motivation, executive functions

## Abstract

Apathy is commonly defined as a loss of motivation leading to a reduction in goal-directed behaviors. This multidimensional syndrome, which includes cognitive, emotional and behavioral components, is one of the most prevalent neuropsychiatric features of Parkinson’s disease (PD). It has been established that the prevalence of apathy increases as PD progresses. However, the pathophysiology and anatomic substrate of this syndrome remain unclear. Apathy seems to be underpinned by impaired anatomical structures that link the prefrontal cortex with the limbic system. It can be encountered in the prodromal stage of the disease and in fluctuating PD patients receiving bilateral chronic subthalamic nucleus stimulation. In these stages, apathy may be considered as a disorder of motivation that embodies amotivational behavioral syndrome, is underpinned by combined dopaminergic and serotonergic denervation and is dopa-responsive. In contrast, in advanced PD patients, apathy may be considered as cognitive apathy that announces cognitive decline and PD dementia, is underpinned by diffuse neurotransmitter system dysfunction and Lewy pathology spreading and is no longer dopa-responsive. In this review, we discuss the clinical patterns of apathy and their treatment, the neurobiological basis of apathy, the potential role of the anatomical structures involved and the pathways in motivational and cognitive apathy.

## 1. Introduction

Parkinson’s disease (PD) is a neuropsychiatric condition that combines a broad range of motor and non-motor signs from the prodromal stage [1,2,3]. Beyond the classic motor triad of akinesia, rigidity and resting tremor, PD is also accompanied by several behavioral and/or neuropsychiatric syndromes including apathy and impulse control disorders (ICDs) [4,5]. These disorders significantly impair the quality of life of patients and their caregivers, and are therefore a crucial issue in the diagnosis and management of PD [6,7]. Apathy, one of the most frequent and debilitating behavioral signs of PD, has been defined by the International Society for Central Nervous System Clinical Trials Methodology (ISCTM) as a quantitative and persistent reduction in goal-directed behaviors (GDBs), resulting in a significant impairment to patients’ daily life functioning [8,9]. This working group specified that symptoms should affect at least two of the three following dimensions of apathy: (i) diminished initiative, (ii) diminished interest and (iii) diminished emotional responsiveness. The prevalence of apathy increases along with disease progression [10]. In the early stage, the apathy–anxiety–depression triad mirrors the motor triad of PD [4,5,11]. During the motor and non-motor fluctuations stage, apathy is commonly observed in the “OFF” periods and in patients who have undergone chronic, bilateral deep brain stimulation (DBS) of the subthalamic nucleus (STN) [5,11,12]. In both the early and fluctuation stages, apathy may be considered as a disorder of motivation that is to some extent dopa-responsive. Motivation is defined by the drive toward a goal that is elicited by environmental or internal stimuli [13,14]. Motivational apathy is embodied by the so-called reward deficiency syndrome and underpinned by combined dopaminergic (DA) and serotonergic denervation within the mesocorticolimbic (MCL) pathway [5,15,16]. The role of chronic, bilateral STN-DBS in apathy is controversial. Some authors argue that STN-DBS has a psychostimulant effect, while others argue in favor of STN-DBS-induced apathy [12,17,18,19,20]. In the late stage of PD, apathy may be associated with cognitive decline, dysexecutive syndrome and PD dementia. Cognitive apathy may reflect widespread brain damage including Lewy pathology spreading and the concomitant involvement of other neurotransmitter systems, including cholinergic and noradrenergic circuits, and is no longer dopa-responsive [21,22,23,24,25]. In sum, apathy is a multidimensional neuropsychiatric syndrome that combines motivational and cognitive deficits and results from a complex interaction between PD, neurotransmitter dysfunction and therapeutic management [4,5,11,12]. In this review, we aimed to describe apathetic syndromes, their clinical and anatomical correlates and the therapeutic options throughout PD progression. 

## 2. Prevalence and Clinical Correlates

The reported prevalence of apathy in PD greatly varies across studies. These discrepancies may be explained by several factors including population characteristics, PD stage, assessment tools and therapeutic management. Overall, apathy has a prevalence of around 40% in PD for all stages [26]. It is estimated to affect around 30% of de novo PD patients, and prevalence increases along with PD progression up to 50% in the late stage [6,10,26,27]. 

Although apathy sometimes appears in isolation, motivational apathy frequently co-occurs with other neuropsychiatric signs including anhedonia, anxiety, depression and fatigue from the early stage of PD [28,29,30,31,32]. Apathy, however, is distinct from depression, as it is not associated with feelings of sadness, worthlessness, hopelessness, guilt or self-blame [28]. On the contrary, anhedonia—defined as a decreased ability to anticipate and/or experience pleasure—is a common dimension of both motivational apathy (anticipatory anhedonia) and depression (consummatory anhedonia) [30,33,34]. Interestingly, a recent data-driven analysis of 193 de novo PD patients exhibited three distinct clusters of PD and distinguished the neuropsychiatric phenotype, which comprised apathy, anxiety and depression as its core dimensions [35], and which may also be encountered in PD patients treated with chronic STN-DBS. In addition, fatigue that has both subjective (symptomatic) and objective (fatigability) dimensions is also frequently associated with motivational apathy and/or depression in PD [36,37,38,39,40]. Indeed, fatigue, one of the most common complaints in PD, accompanies neuropsychiatric signs from the prodromal stage of PD and is correlated with apathy, anxiety and depression [31,37,38,40,41]. Additionally, a lack of motivation, a key feature of apathy, is a predictor of fatigue in PD [30]. In light of the clinical overlap between these neuropsychiatric signs, it has been suggested that motivational apathy, anxiety and depression could define the so-called hypodopaminergic behavioral syndrome and that fatigue could expand this neuropsychiatric triad of PD [42,43]. 

In advanced PD, cognitive apathy is associated with executive function impairment, cognitive decline and dementia [21,28,44]. Poorer performance in frontal neuropsychological batteries has been shown in apathetic patients compared to non-apathetic patients [21,28,31]. Moreover, a higher rate of dementia has been found in apathetic patients compared to non-apathetic and non-depressed patients [21,28,44].

## 3. Cognitive, Anatomo-Clinical and Computational Models of Apathy

Several models have been developed to account for apathy and motivated disorders in neuropsychiatry. These models include cognitive, anatomo-clinical and computational approaches. Following the pioneering work of Marin [45], and assuming a close interplay between limbic, cognitive and motor striato-thalamo-cortical circuits, Brown and Pluck conceptualized apathy as a deficit of motivation and GDBs [46]. They distinguished several different steps between intention to act and action achievement, including sensitivity to the valence of internal or external stimuli, internal representation of reward, selection, initiation, control of appropriate actions, inhibition of inappropriate actions and execution [46]. In addition, they put forward the role of reward satisfaction in maintaining the causal link between internal state, action and reward [46]. According to this model, GDBs are elicited by an a priori knowledge of the contingency between action and outcome, and are directed toward obtaining the reward [46] in line with the concept of reward deficiency syndrome [5]. In this context, apathy has been conceptualized as a failure to achieve voluntary actions and purposeful GDBs. Using this model and the anatomo-clinical method, Levy and Dubois categorized apathetic syndromes into three subtypes: (i) emotional–affective apathy (i.e., motivational apathy), related to alterations in limbic circuits, (ii) cognitive apathy, related to dysfunction in associative circuits (i.e., cognitive apathy) and (iii) self-activation deficits, combining alterations within limbic and associative circuits [47].

Recently, behavioral neuroscience has given rise to the concept of effort-based decision making (EBDM) to obtain rewards in order to account for motivated behavior disorders, including motivational apathy [48,49,50]. The EBDM model posits a crucial role of the valuation system, which calculates a cost/benefit ratio based on the effort required to initiate and achieve a goal [48,50]. According to this model, motivational apathy may result either from reduced sensitivity to rewards or increased sensitivity to effort costs. In both cases, it leads to the disruption of the initiation, vigor and persistence of actions in the pursuit of rewards [13,48]. The EBDM model is in accordance with the model of fatigue developed by Chaudhuri and colleagues, which postulates that motivational input, effort and perceived effort are the main determinants of fatigue [36,51]. As such, decreased reward sensitivity and/or increased effort costs could account for the co-occurrence of apathy and fatigue in PD, with fatigue being a very frequent complaint that may lead to the identification of apathy [43]. In the same way, negative affect, which characterizes depression, could alter the cost/benefit ratio and increase perceived effort, thereby accounting for the co-occurrence of depression, fatigue and apathy. Anhedonia could be a link between apathy, depression and fatigue, since anticipatory anhedonia and consummatory anhedonia are associated with apathy and depression, respectively [34]. 

In recent decades, animal research has been helpful in improving our knowledge of the mesolimbic dopamine system’s role in effort-related motivation, including effort expenditure during instrumental behavior tasks and EBDM [13,48,52,53]. EBDM studies typically involve offering animals a choice between a high-effort, preferred reinforcer and a low-effort, less-preferred reinforcer. The main experimental paradigms used are operant tasks where animals choose between pressing a lever a fixed number of times to obtain preferred food or freely accessing less rewarding standard food [13,48,52,53]. Alternatively, the T-Maze barrier procedure where two cost/benefit options (i.e., a high-effort, preferred option and a low-effort, less-preferred option) are presented in two choice arms has been used. Most research on this topic focuses on the role of DA systems, particularly the nucleus accumbens (NAc). In rodent models, DA antagonists, when administered systemically or directly into the NAc, consistently induce a bias toward selecting the low-effort, low-reward option [13,48,52,53]. However, when effort costs were equalized, DA depletion neither affected reinforcer preference nor impaired motor capacity, meaning that reduced willingness to allocate effort for rewards was the driving force responsible for behavioral changes [13,48,52,53]. The mesolimbic DA system is part of a distributed forebrain circuit, including the basolateral amygdala, prefrontal/anterior cingulate cortex and ventral pallidum. Anatomical lesions in these sites produce similar changes in choice preference as DA manipulations [13,48]. Other neurotransmitters and neuromodulators, such as acetylcholine, adenosine, serotonin and gamma-aminobutyric acid (GABA), also play a role in regulating effort-related choice [13,48]. Elucidating the contributions of each component within this circuitry is challenging but also offers opportunities for the development of innovative pharmacological strategies. In this context, the utilization of animal models proves to be an insightful and valuable resource.

## 4. Diagnosis and Assessment

Numerous scales are currently used in clinical practice to assess apathy, including auto-questionnaires such as the Apathy Scale (AS) [54], the Apathy Evaluation Scale (AES) [55] and the Apathy Inventory (AI) [56]. Other tools used to assess apathy are semi-structured interviews performed by trained neuropsychologists, namely the Neuropsychiatric Inventory (NPI) [57], the Lille Apathy Rating Scale (LARS) [58] and the Ardouin Scale of Behaviors in Parkinson’s Disease (ASBPD) [42,59]. To date, only the AS has fulfilled the criteria to be recommended by the Movement Disorder Society (MDS) to assess apathy in PD [29]. The apathy item of UPDRS has been validated for the screening of apathy [29]. LARS and ASBPD are specifically designated for PD. LARS investigates the subtypes of apathy including the motivational, cognitive and auto-activation components in accordance with the models of apathy described above [46,47,58]. The ASBPD is the only scale that assesses the whole spectrum of motivated behaviors of PD, including hypodopaminergic behaviors, hyperdopaminergic behaviors and non-motor fluctuations [42,59]. 

## 5. Corticobasal Circuits and Motivated Behaviors

**Figure 1:**
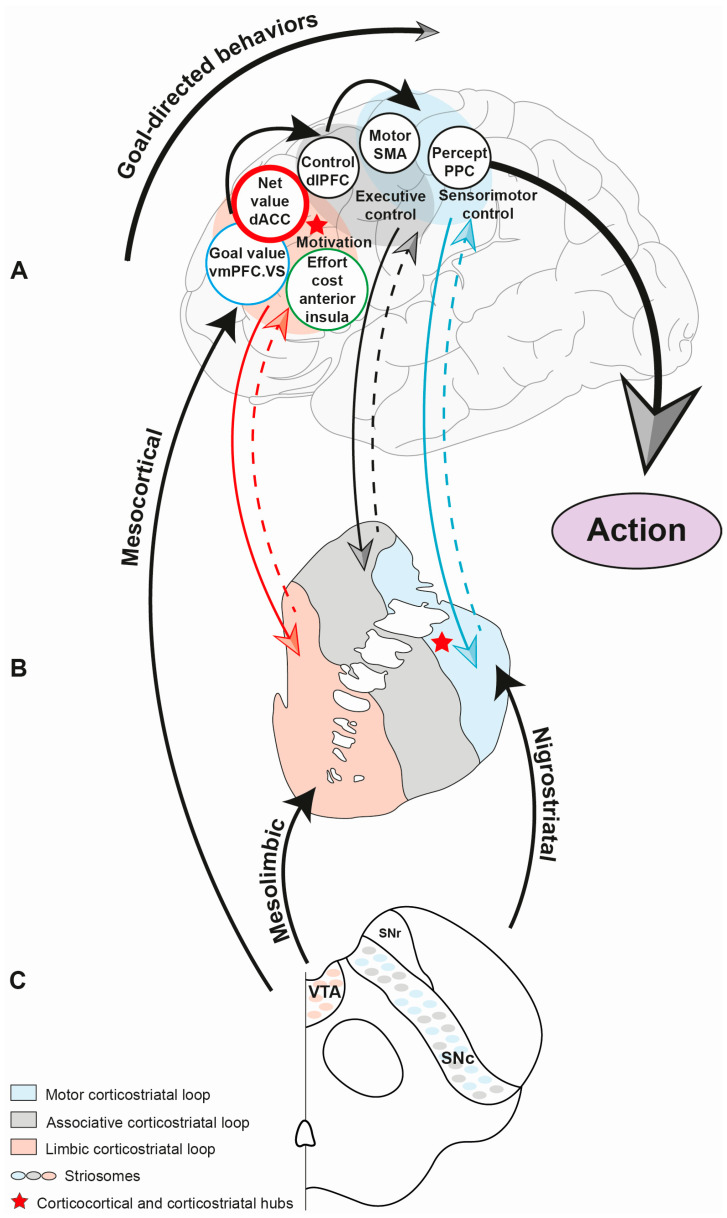
Motivation, cognitive and premotor systems involved in human apathy. The motivation system (i) computes the net value between the goal, the cost/benefit and effort, (ii) is underpinned, in the case of apathy, by dysfunction within the mesocorticolimbic pathway and (iii) is embodied by motivational apathy and the amotivational behavioral syndrome of PD. The cognitive system (i) is involved in the planning and execution of action, (ii) is underpinned, in the case of apathy, by dysfunction within the dorsolateral prefrontal cortex and caudate nucleus within the cortico-striato-thalamo-cortical circuits, and (iii) is embodied by cognitive apathy. The premotor system (i) energizes movement and (ii) is underpinned, in the case of apathy, by dysfunction within the motor cortico-striato-thalamo-cortical circuit. (**A**) Hierarchical control of goal-directed behaviors within the cortex. (**B**) Segregated pathway within the striatum. (**C**) Dopaminergic projections. SNc: substantia nigra pars compacta, STN: subthalamic nucleus, VTA: ventral tegmental area.

The basal ganglia (BG) are paired anatomical structures found in the deep gray matter. They are comprised of the putamen and the caudate nucleus (which together constitute the striatum), the globus pallidus (which contains an internal and external part), the subthalamic nucleus and the substantia nigra [60]. The BG convey motor, cognitive and emotional information via complex, parallel and segregated cortico-subcortico-cortical circuits [60,61,62,63]. Axonal tracing experiments in rodents and non-human primates have shown evidence for parallel and integrated networks within these corticobasal loops [62,63]. These studies have highlighted the specific prefrontal cortical projections to specific parts of the striatum. Three functional corticostriatal circuits were identified: the sensorimotor circuit, which connects the premotor areas with the putamen, the associative circuit, which links the dorsolateral prefrontal cortex (DLPFC) with the caudate nucleus, and the limbic circuit, which connects the orbitofrontal (OFC) and anterior cingulate (ACC) cortices to the ventral striatum (VS) [62,63]. Importantly, Haber and colleagues demonstrated the crucial role of midbrain DA nigrostriatal and mesolimbic projections in processing information circulating in the loops [64]. Moreover, these authors hypothesized a spiral pattern of striato-nigro-striatal projections connecting the ventral striatum to cognitive and motor areas, thereby integrating this information [62,64]. Recently, neuroimaging studies using resting state functional MRI and diffusion tractography have confirmed segregated and overlapping connections, supporting the hypothesis of parallel and integrated networks within these corticostriatal circuits in humans [65,66,67,68,69]. Indeed, connectional hubs that integrate motor, cognitive and motivational inputs, and that modulate striatal processing, have been shown [62,63]. In particular, the convergence of reward-related and cognitive projections from the ACC, OFC and DLPFC to striatal (corticostriatal) hubs and also to rostral anterior cingulate (corticocortical) hubs has been demonstrated [62,63]. These segregated and integrated pathways provide the anatomical substrate for GDBs and EBDM in humans (Figure 1). Disruption within these networks may underlie motivated disorders, including apathy.

Lesion-based rodent and non-human primate models have confirmed the causative role of DA depletion in both the mesocorticolimbic and the nigrostriatal pathways in the development of behavioral impairments related to apathy [70]. As previously mentioned, Dopamine antagonists, when administered systemically or directly into the NAc, or anatomical lesions within the NAc, consistently resulted in a low-effort bias [53,71]. Moreover, these results have been corroborated by recent studies using optogenetic, chemogenetic and physiological techniques [71]. Notably, Fischbach-Weiss and colleagues demonstrated that optogenetic inhibition of the VTA DA neurons in TH-IRES-Cre mice suppressed both the initiation and maintenance of effortful instrumental responding [71,72]. By injecting neurotoxin 6-hydroxydopamine (6-OHDA) into discrete areas of the SNc or VTA in rats, Carnicella and colleagues aimed to disentangle both the mesocorticolimbic and the nigrostriatal DA pathways in motivational processes [70,73]. These lesion-based models exhibited distinct DA denervation patterns and DA loss within either the dorsal (70%) or ventral (40–60%) striata. Importantly, denervation within the dorsal striatum below 80% guaranteed, according to the authors, the absence of significant motor impairment [70,73]. Partial DA denervation induced by a bilateral stereotaxic infusion of 6-OHDA into the SNc dramatically impaired motivational preparatory processes evaluated in an operant conditioning paradigm without affecting the rewarding/reinforcing properties of the reinforcers used [74,75,76]. A marked motivational deficit was observed specifically when an instrumental preparatory action was required, a behavioral phenotype that appears to be highly reminiscent of at least some forms of apathy [5,47,77]. Surprisingly, none of the motivational deficits described above were observed with partial and bilateral 6-OHDA lesions affecting the medial VTA [70,73]. Furthermore, only a nearly complete loss of DA within the MCL DA pathway resulted in apathy. Thus, this striking dissociation between the behavioral effects of a partial DA lesion of the VTA or the SNc strongly supports the prominent role of the nigrostriatal system in motivational processes and apathy [70,73]. 

Previous research conducted in non-human primates has demonstrated a causal link between BG circuit dysfunctions and motivated behavior disorders [78,79,80,81]. In particular, Worbe et al. found that the local injection of bicuculline (a GABA antagonist) within the VS induced diminished behavioral activity that mimicked an apathetic state [80]. Furthermore, two studies in 1-methyl-4-phenyl-1,2,3,6-tetrahydropyridine (MPTP)-lesioned non-human primates have highlighted the key role of the mesolimbic and mesocortical DA pathways in the willingness to attempt goal-directed behaviors [82,83]. In these studies, PET imaging changes and DA cell loss within the mesolimbic and mesocortical pathways predicted apathetic behaviors better than metabolic changes and DA cell loss within the nigrostriatal pathway did [82,83].

Altogether, these preclinical data in animal models strongly suggest a close interaction between the mesocorticolimbic and nigrostriatal pathways in the pathophysiology of motivated behaviors and PD apathy. The spiral pattern of striato-nigro-striatal projections connecting the ventral striatum to cognitive and motor striatum areas, although not yet demonstrated in humans, may offer the anatomical substrate connecting the mesolimbic and mesocortical pathways to the nigrostriatal pathways, thereby intimately linking motivation to action [62,63].

In humans, pioneering lesion studies have demonstrated an apathetic state in cases of damage to the caudate nuclei, medial pallidum and mediodorsal thalamic nuclei [84,85,86]. Importantly, the authors made a causal link between apathy and a “prefrontal-like” syndrome, thus stressing the role of BG and prefrontal cortex disconnection in apathy [47]. In recent years, structural and functional neuroimaging studies carried out in PD, other neurodegenerative disease and stroke patients have improved the understanding of the anatomical correlates of apathy [87,88]. Overall, these studies have demonstrated disruption within the dorsal ACC, VS, thalamus and connected brain regions, including the OFC for motivational apathy and dorsomedial and DLPFC for cognitive apathy. Finally, a recent neuroimaging study by Biondetti and colleagues analyzed relationship and temporal changes between the DA system and iron metabolism in PD [89]. By using neuromelanin-sensitive and iron-sensitive MRI and dopamine transporter positron emission tomography (PET), the authors showed that striatal DA denervation within the sensorimotor, associative and limbic striata began decades before disease diagnosis, followed by abnormal iron metabolism and finally neuromelanin changes in the substantia nigra pars compacta, which occurred according to the same spatial and temporal gradient as the DA denervation [89]. Hence, these results support the involvement of both the nigrostriatal and mesocorticolimbic pathways in PD from the early stage. Taken together, these data in humans strengthen the link between the limbic and cognitive corticobasal circuits’ dysfunction and apathy.

## 6. Neurobiology of Apathy

### 6.1. Dopamine, Reward and Effort

**Figure 2:**
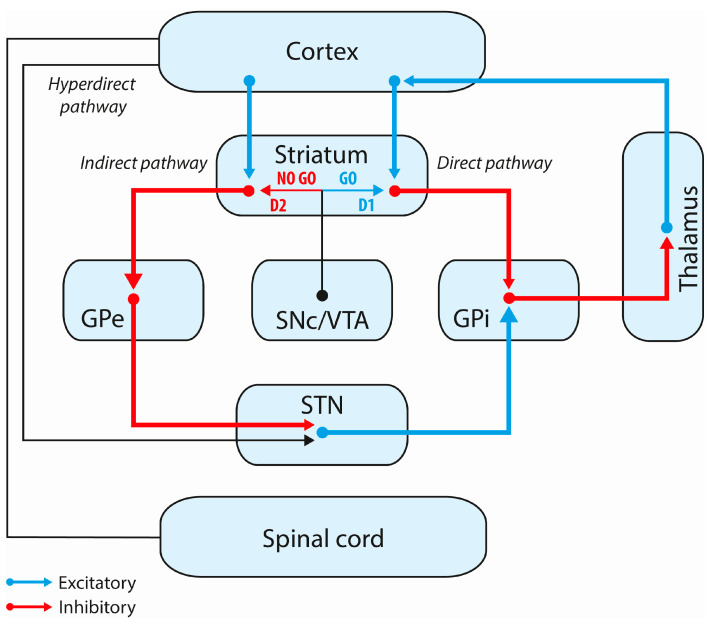
Model of the basal ganglia. Cortico-striato-thalamo-cortical circuits are separated into two distinct pathways: the direct (GO) pathway and the indirect (NO GO) pathway. The “GO” pathway leads to disinhibition of the thalamus and facilitates execution of action. The “NO GO” pathway leads to inhibition of the thalamus and suppresses action. Dopamine projects from the SNc/VTA to striatal medium spiny neurons and modulates these two pathways via D1 and D2 receptors. GPe: globus pallidus externus, GPi: globus pallidus internus, SNc: substantia nigra pars compacta, STN: subthalamic nucleus, VTA: ventral tegmental area.

Dopamine plays a crucial role in reward-based behaviors and reinforcement learning. A seminal work by Schultz showed that dopamine encodes the reward prediction error in non-human primates [90,91]. In the case of better-than-expected reward, a phasic secretion of dopamine occurs, whereas in the case of an absence of expected reward, a transient dip happens. Some other works advocate that dopamine modulates reward-based behaviors via striatal projections [92,93,94]. From this understanding, approach behaviors are mediated by the direct pathway (“GO pathway”) modulated via D1 receptors (D1Rs), whereas avoidance behaviors involve the indirect pathway (“NO GO pathway”) modulated via D2 receptors (D2Rs) (Figure 2). 

Aside from the roles of D1Rs and D2Rs in modulating the direct “GO” and indirect “NO GO” pathways, it has been suggested that dopamine D3 receptors (D3Rs) also play a critical role in motivated behaviors and Parkinsonian apathy [73]. Indeed, D3Rs, which are mainly expressed within the limbic system, are involved in a large spectrum of neuropsychiatric disorders, including schizophrenia and drug addictions [95]. Interestingly, some preclinical and clinical data advocate for their crucial role in motor and behavioral signs of PD [95]. D3Rs are downregulated following DA lesions in rats and monkeys [96,97], but also in drug-naïve PD patients [98]. Moreover, in their 6-OHDA rat model of Parkinsonian apathy, Carnicella and colleagues discovered a selective decrease in D3R expression in the dorsal striatum of lesioned rats [70,73]. Furthermore, the inhibition of D3R neurotransmission in non-lesioned animals was sufficient to reproduce the motivational deficit observed in lesioned rats [70,73]. Interestingly, the D2/D3Rs agonists ropinirole and pramipexole effectively reversed the motivational deficit induced by the lesion, highlighting this receptor as a promising target for the treatment of motivational deficits [70,73]. In line with this, pharmacological studies targeting D2/D3Rs in PD patients showed an improvement in postoperative apathy after the administration of either ropinirole or piribedil [99,100]. 

Additionally, dopamine has been found to play a role in reward sensitivity in humans. A decrease in reward sensitivity was found during two oculomotor decision making tasks in a patient who suffered from bilateral pallidal lesions [101]. Interestingly, reward sensitivity was restored via the introduction of either levodopa or a dopamine agonist. Similar results have been shown in fluctuating PD patients in the “OFF” and “ON” state [102]. Finally, a decrease in reward sensitivity has been demonstrated in PD motivational apathy [103]. Other works in both rodents and humans have investigated the role of dopamine in integrating the cost–benefit ratio [48,49,50,52,71,104]. According to the EBDM model, DA depletion in mice favored low effort for low reward [71]. In contrast, dopamine in rodents and PD patients biased the choice toward large effort for greater rewards, as predicted by the model [48,71,104]. Although still debated, the role of dopamine has been reported by some authors to be prominent in reward processing as compared to effort valuation [105,106]. 

### 6.2. The Dopaminergic Behavior Continuum Hypothesis

Some authors have suggested that motivated disorders in PD may be considered as a continuum of clinical and pathophysiological behaviors, ranging from “hypodopaminergic” to “hyperdopaminergic” behaviors [107,108]. According to this hypothesis, motivational apathy, anxiety and depression embody “hypodopaminergic behaviors”, while the set of behavioral addictions (such as hypersexuality, compulsive shopping, gambling, binge eating, cyberaddiction, punding) related to impulse control disorders (ICDs) embody “hyperdopaminergic behaviors” [42,107]. The hypothesis is based on clinical and neurophysiological data found in de novo PD patients and PD patients on dopamine replacement therapy (DRT), but also in patients who have undergone bilateral STN-DBS [15,92,100,109]. Based on Frank’s model, Bódi et al. investigated reward and punishment processing as well as personality traits in de novo PD [92,109]. Using a feedback-based probabilistic learning task, these authors showed that patients with unmedicated de novo PD had predominantly harm-avoidance personality traits and a learning curve elicited by punishment avoidance rather than by reward seeking [92,109]. Importantly, deficits in reward processing and personality traits were reversed by dopamine agonists [92,109]. Some data from de novo PD and STN-PD patients before and after surgery strengthen the DA behavior continuum hypothesis. A recent data-driven analysis identified the neuropsychiatric cluster, which combines apathy, anxiety, depression and harm-avoidance, as one of the main endophenotypes observed in de novo PD [35]. Interestingly, this phenotype is close to the DA behavioral withdrawal syndrome encountered in some STN-DBS PD patients during postoperative follow-up, but is the exact opposite of the one encountered in the preoperative period [110]. These findings have been deeply investigated by two studies in STN-DBS PD patients [15,100]. During the pre-operative period, one third of PD patients exhibited an appetitive functioning mode, characterized by a high proportion of non-motor fluctuations but also hyperdopaminergic behaviors, echoing motor fluctuations and dyskinesias, respectively [15]. After surgery, the motor benefit of chronic bilateral STN-DBS was accompanied by a drastic reduction in DA drugs followed by a significant decrease, not only in motor fluctuations and dyskinesia, but also in non-motor fluctuations and hyperdopaminergic behaviors. However, nearly half of the patients developed DA behavioral withdrawal syndrome mainly expressed by the hypodopaminergic apathy–anxiety–depression triad [15]. Furthermore, using [11C]-raclopride PET before and after a methylphenidate challenge, these authors demonstrated that postoperative motivational apathy was underpinned by DA denervation within the MCL pathway, including the left and right amygdala, the bilateral OFC and the dorsolateral, posterior cingulate and temporal cortices [15]. In addition, in a 12-week prospective, randomized, double-blind controlled trial of 37 patients with apathy, the authors showed that postoperative apathy and depression responded to piribedil, a D2/D3 dopamine agonist, while a trend toward significance was noticed for anhedonia [100]. Finally, two other [11C]-raclopride PET studies in PD patients with behavioral addictions showed a decrease in binding potential within the MCL pathway, namely the VS, after levodopa intake or during a gambling task [111,112]. Altogether, these studies support the hypothesis of a clinical and pathophysiological continuum from hypodopaminergic to hyperdopaminergic behaviors, reflecting denervation (in unmedicated de novo PD), sensitization (in PD patients who are candidates for STN DBS) and desensitization (DA behavioral withdrawal syndrome) within the MCL pathway [113,114,115,116]. Sensitization is the result of DA denervation—which is related to PD spreading—combined with long-lasting pulsatile DRT. It is associated with: (i) dopamine level fluctuations in the synaptic cleft, resulting in motor and neuropsychiatric fluctuations and (ii) the upregulation of postsynaptic receptors, resulting in dyskinesia and ON-euphoria, which promotes impulse control disorders and behavioral addictions [113,114,115,116]. Conversely, desensitization is associated with a marked reduction in DA medication for weeks to months following bilateral chronic DBS. This leads to a decrease in dyskinesia but also to behavioral withdrawal syndrome including apathy, anxiety and depression, which is accompanied by a decrease in the magnitude of neuropsychiatric fluctuations [113,114,115,116]. The DA behavior continuum hypothesis is also supported by Dagher et al., who postulated a model based on a multidirectional control of approach and avoidance behaviors via tonic stimulation in the form of striatal DA projections [93,94]. However, some data challenge the DA behavior continuum hypothesis. For example, the study by Frank et al. showing a close relationship between PD pathology, DRT and reward-based learning has not been replicated [92,117]. Moreover, a recent randomized controlled double-blind study failed to demonstrate the efficacy of rotigotine on apathy in a de novo PD cohort, which the authors attributed in part to a strong placebo effect [118]. Furthermore, recent studies on large PD and healthy subject cohorts have demonstrated the co-occurrence of apathy and impulsivity, which contradicts the hypothesis of one clinical DA continuum between these behaviors [119,120]. In the same way, other neurotransmitter systems are involved in PD apathy, particularly serotonergic, noradrenergic and cholinergic circuits [16,22,23,24,25,121,122,123,124]. 

### 6.3. The Role of the Subthalamic Nucleus

The STN is commonly divided into motor, associative and limbic parts. Although still a matter of debate, this tripartite anatomical subdivision has been recently confirmed in humans [68,69,125]. The role of the STN in motor and cognitive control has been well documented. Notably, a seminal work from Frank et al. highlighted the crucial role of the STN in response selection and decision making [126]. The STN receives excitatory inputs from the premotor cortex and the so-called “hyperdirect pathways” and projects excitatory outputs to both the globus pallidus internus (GPi) and globus pallidus externus (GPe) (Figure 2) [126]. Furthermore, some evidence in rodents, primates and humans suggests a key role of STN in motivated behaviors. In essence, by injecting anterograde tracers into the prefrontal cortex of macaques, Haynes et al. showed that the hyperdirect pathway could be extended to motivational and cognitive brain regions [127]. Specifically, they showed connections between the STN and the ventral pallidum but also between the STN and the ventromedial prefrontal cortex (vmPFC), OFC and ACC, suggesting a key role of the STN in reward processing [127]. In line with this, studies in rodents advocated for the role of STN neurons in coding reward expectation and reward prediction errors [128,129]. Recently, Nougaret et al. recorded the activity of STN neurons in two male monkeys performing a visuomotor motivational task, in which visual cues indicated the amount of force required to obtain the related amount of reward [130]. Interestingly, they evidenced the existence of force- and reward-modulated neurons. After the occurrence of the visual stimuli, the force-modulated neurons mainly fired when high effort was required. Differently, the activity of the population of reward-modulated neurons encoded the motivational value of the stimuli. Both populations could play complementary roles, one in the implementation of the difficulty of the action and the other in enhancing or slowing its execution based on the subjective value of the reward [130]. Altogether, these studies are consistent with the role of the STN in computing reward and cost/benefit value. Other studies investigated the effects of STN-DBS on motivated behaviors. Vachez and colleagues showed that STN-DBS could induce a motivational deficit in naïve rats and exacerbate a motivational deficit in a rodent model of PD [131]. Importantly, in both cases, a loss of motivation was fully reversed via chronic treatment with pramipexole, a D2 and D3 DA receptor agonist [131]. Moreover, the same group showed that STN-DBS induced changes in the expression of DA receptors after prolonged unilateral stimulation (4 h) in intact rats and in rats with total DA denervation [132]. STN-DBS increased D1Rs levels in almost all of the striatal areas examined, in both intact and denervated rats. In contrast, STN-DBS led to a large decrease in D2Rs and D3Rs levels, limited to the NAc and independent of the DA state of the animals [132]. Finally, the same group performed a systematic analysis of rodent studies, assessing the effects of STN-DBS on reward seeking, reward motivation and reward consumption across a variety of behavioral paradigms [133]. They found that STN-DBS consistently decreased reward motivation, seeking and consumption across a variety of behavioral models. These data provide experimental evidence that chronic STN-DBS by itself can induce a loss of motivation in rats. In the near future, optogenetic tools could be used to establish causal links between DBS effects on STN microcircuitry and motivation deficits [134].

Despite broad agreement on motor efficacy, the role of bilateral chronic STN-DBS on motivated behaviors is highly debated in humans [12]. Some studies have highlighted psychotropic dopa-like effects, including mirthful laughter and hypomania, while others advocate for STN-DBS-induced apathy [17,18,19,20]. Most of these studies have been uncontrolled and have not assessed the precise location of the active contacts, nor modeled the volume of tissue activated (VTAc). In addition, the postoperative management of DRT may have biased the behavioral effects attributed to STN-DBS. Moreover, the effect of the acute onset of STN-DBS should be distinguished from the effect of chronic STN-DBS on motivation. Recently, the development of an imaging toolbox has helped to visualize the active contacts of stimulation, but also to model VTAc and quantify the overlap between VTAc and the three anatomo-functional parts of the STN [135]. Overall, these studies support the hypothesis of a prominent role of chronic stimulation of the STN in the occurrence of behavioral manifestations, but again with conflicting results. Some studies found a higher risk of postoperative apathy in the case of stimulation of either the motor or the limbic parts of the STN or the zona incerta (ZI), whereas others posited a psychostimulant effect and a higher risk of postoperative mania in the case of stimulation within the limbic part of the STN [136,137,138,139,140]. The latter results called into question the reality of a “motivational hotspot” in cases of stimulation of the limbic part of the subthalamic nucleus, comparable to the “motor hotspot” that has been suggested in the case of stimulation of the sensorimotor part of the STN [141].

### 6.4. Motivational and Cognitive Apathy

**Figure 3:**
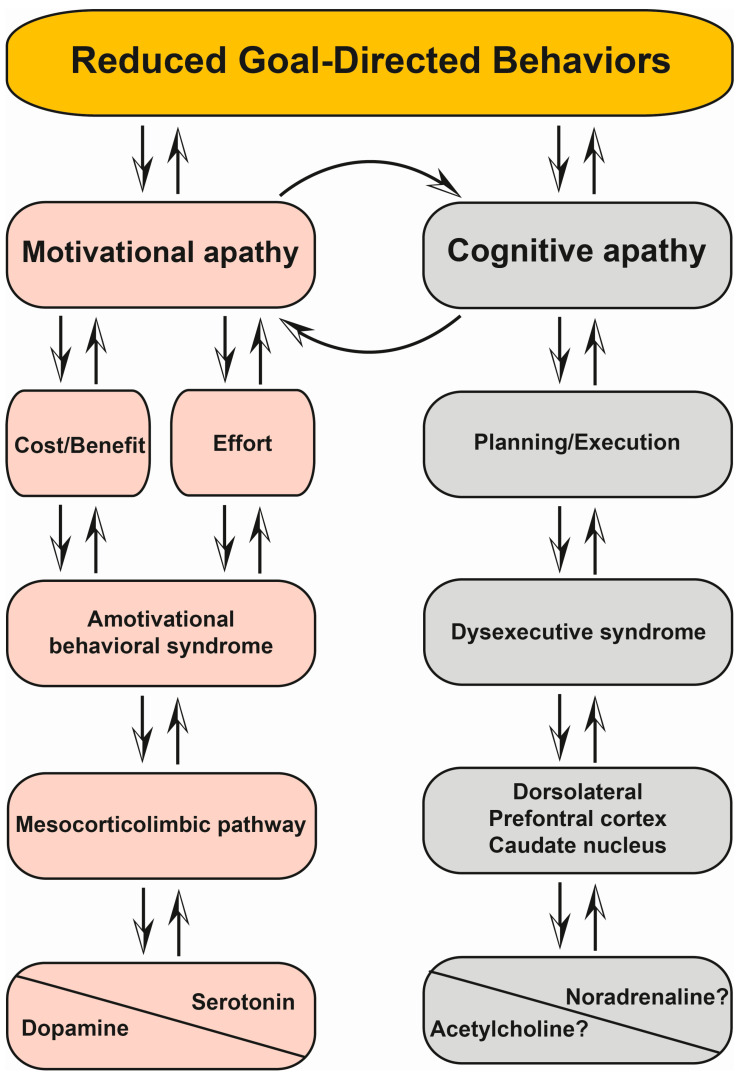
Mechanistic, clinical and pathophysiological features of motivational and cognitive apathy.

Motivation, as defined above, is the drive toward a goal that is elicited by environmental or internal stimuli [13,14]. Cognitive control can be defined as a set of executive mechanisms required for achieving the goal, once selected [142]. Motivation and cognitive control are the two main components of GDBs [47]. Thus, the dysfunction of either motivation or cognitive control may lead to reduced GDBs, resulting in motivational and cognitive apathy, respectively (Figure 3) [47]. 

Cognitive control involves different regions of the prefrontal cortex, which provide hierarchical control of GDBs via “top-down” projections. These projections are organized along a rostro-caudal gradient of abstraction from the higher-level task control in rostral prefrontal corticostriatal circuits to the lower-level task processing in caudal sensorimotor corticostriatal loops [143]. Interestingly, a recent study demonstrated the role of “bottom-up” striatal dopamine projections in computing the cost/benefit ratio of cognitive work, thereby promoting the willingness to exert cognitive effort [144]. Moreover, as mentioned above, previous neuroanatomical studies in non-human primates have shown a spiral organization of striato-nigro-striatal DA projections connecting the limbic ventromedial part of the striatum to its cognitive and motor dorsolateral parts [61,62,63,64]. Hence, striatal DA projections would enhance approach behaviors in biasing the cost/benefit ratio of cognitive effort toward a higher sensitivity to the benefits and lower sensitivity to the costs, thereby facilitating the allocation of cognitive resources to achieve GDBs. Together, these data suggest a close relationship between the motivational (the drive toward the goal) and cognitive (the ability to achieve the goal) components of GDBs. Therefore, apathy could result either from a lack of motivation to engage in cognitive effort (motivational apathy), or from a failure to mobilize cognitive resources (cognitive apathy) (Figure 3). Motivational apathy could be embodied by the so-called reward deficiency syndrome, which is underpinned by combined and widespread DA and serotonergic denervation within the MCL pathway [5,15,16,123]. Indeed, the role of serotonergic denervation within the MCL pathway has been shown in motivational apathy from the early stage of PD [16,124]. Specifically, these authors suggested that serotonergic circuits may compensate for DA denervation and that combined DA and serotonergic denervation within the MCL pathway may underlie motivational apathy in PD [122]. In this later study, Prange and colleagues studied the association between the longitudinal evolution of neuropsychiatric signs and DA and serotonergic innervation in 13 apathetic and 13 non-apathetic de novo PD patients at baseline and up to five years after diagnosis [122]. They used [11C]PE2I and [11C]DASB PET imaging tracers with highly specific binding to dopamine transporters (DATs) and serotonin transporters (SERTs), respectively [122]. The authors showed that changes in [11C]DASB BP_ND_ in the ACC were negatively correlated with apathy score, indicating greater serotonergic innervation relative to baseline when apathy improved [122]. Moreover, PD patients who reverted to baseline apathy exhibited an increase in or preservation of [11C]DASB BP_ND_ in the ACC in comparison with the healthy controls [122]. Importantly, the progression of nigrostriatal and mesocorticolimbic DA denervation was similar between the two groups [122]. The authors concluded that serotonergic plasticity had contributed to the reversal of apathy along with DRT, suggesting a compensatory mechanism [122]. Moreover, in line with previous research in MPTP-lesioned monkeys, the authors hypothesized the collateral sprouting of serotonergic fibers within the limbic system as the mechanism responsible for this compensation, since ectopic serotonergic terminals would have the molecular machinery to covert levodopa into dopamine and to release dopamine within the MCL pathway [122,145]. This complex interaction between DA and serotonergic systems may account for some discrepancies in pharmacological studies, examining the effect of both DA and serotonergic drugs in PD apathy [27].

Accordingly, motivational apathy may predominate in de novo PD but also in dopamine behavioral withdrawal syndrome following STN-DBS surgery, which is dopa-responsive [5,11,12,100]. In line with the DA behavior continuum hypothesis, we propose that motivational apathy, anhedonia, anxiety, depression and fatigue could define the amotivational behavioral syndrome of PD (Figure 3). This proposal is supported by (i) a broad conceptual and clinical overlap between these neuropsychiatric signs from the early stage of PD [30,31,36,146,147], (ii) cognitive and computational models of fatigue and motivated behaviors that emphasize cost/benefit and effort valuation as the main drivers of motivated disorders [48,51], (iii) neuroimaging and metabolic studies that found DA and serotonergic denervation within the MCL pathway [15,16,148] and (iv) clinical improvements in motivational apathy, depression and fatigue with DA treatments, and particularly dopamine agonists with a high affinity for mesolimbic D3 receptors [7]. Finally, amotivational behavioral syndrome, including motivational apathy, corresponds to the hypodopaminergic end of the DA behavioral continuum.

Cognitive apathy, which is dopa-resistant, predominates in advanced PD and may accompany cognitive decline and be an early sign of PD dementia [21]. Some arguments advocate for the role of cholinergic circuits in cognitive apathy: (i) cholinergic denervation is associated with an impairment of attentional and executive functions and predicts cognitive decline [149,150,151], (ii) atrophy of the nucleus basalis of Meynert and cholinergic denervation have both been associated with the occurrence of cognitive decline and behavioral disorders, including apathy and depression [152,153,154] and (iii) a randomized controlled clinical trial showed the benefit of rivastigmine, an anti-cholinesterase drug, in the treatment of apathy in advanced PD [22]. In sum, these data suggest that both motivational “bottom-up” and cognitive “top-down” networks may contribute to the occurrence of apathy. Finally, noradrenergic pathways could also be involved in cognitive apathy [23,24,25]. Ye et al. showed that neurodegeneration of the noradrenergic locus coeruleus correlated with apathy and global cognition scores worsening in PD and progressive supranuclear palsy (PSP) patients [25]. Specifically, a strong correlation has been shown in PSP, where executive dysfunction and cognitive decline predominate [25]. Lastly, some neuroimaging studies have suggested a noradrenergic contribution in the pathophysiology of mild cognitive impairment in PD [23].

Motivational and cognitive apathy, although not mutually exclusive, may be associated with distinct PD endophenotypes reflecting distinct Lewy pathology spreading in various brain regions and neurotransmitter systems [10,155,156]. Thus, animal models that mimic DA neuronal loss and the progressive formation of α-synuclein aggregates in various brain regions are needed. In rodents, PD-like synucleinopathy can be induced by overexpressing wild-type or mutated forms of α-synuclein, using genetically modified animals or viral vector injections, or, alternatively, through the intracerebral administration of preformed α-synuclein fibril(s) (PFFs) [157,158,159]. Based largely on Braak’s observations [160], several attempts have been made to generate propagation models in mice by injecting PFFs into the olfactory bulb and peripheral regions [161]. In these models, various motor alterations have been reported, though the data are not consistent. For example, a model combining preformed human α-synuclein fibrils and adeno-associated virus-mediated overexpression of human α-synuclein has been developed [162], and this combined approach reproduced several cardinal features of the human disease, including Lewy-like synucleinopathy, neuroinflammation and progressive DA cell loss. Interestingly, several studies suggest that enhanced α-synuclein expression in either DA, serotonergic or noradrenergic neurons induces mood disturbances, including depressive-like behaviors, which occur prior to the onset of motor pathology [163]. However, to our knowledge, the specific evaluation of the symptoms associated with apathy in mice has not been investigated in these models (unlike the neurotoxic models [70]), and this constitutes an interesting avenue for research.

## 7. Treatment of Apathy

The pharmacological treatment of apathy may target the DA, serotonergic and cholinergic neurotransmitter systems involved in motivational and cognitive apathy. A recent meta-analysis of seven randomized controlled trials showed that pramipexole significantly improved motivation in PD patients, as assessed using Item 4 of the UPDRS-I [164]. Moreover, post hoc analysis of RECOVER, a randomized controlled trial (RCT), showed that a rotigotine transdermal patch improved the amotivational behavioral syndrome of PD, as assessed via the Non Motor Symptoms Scale (NMS), used as an explanatory outcome in 267 PD patients [165]. The efficacy of dopamine agonists on withdrawal apathy after STN DBS has been shown in a randomized controlled trial [100]. A significant improvement in the Starkstein Apathy Scale was found in patients experiencing motivational apathy who received piribedil compared to those who received the placebo [100]. However, a recent RCT failed to demonstrate the efficacy of rotigotine on PD apathy in a de novo drug-naïve PD cohort [118]. Although the efficacy of methylphenidate, a psychostimulant that acts on the MCL pathway, has been suggested in a subgroup analysis of a randomized controlled trial, evidence remains scarce [166]. The use of serotonergic drugs is controversial, as selective serotonin reuptake inhibitors (SSRIs) have been associated with the worsening of PD apathy [167]. Rivastigmine, an acetylcholinesterase inhibitor, has been shown to improve apathy in PD patients without dementia or depression in an RCT [22].

Non-pharmacological interventions including behavioral therapy, physical exercise and non-invasive brain stimulation have also been considered to treat PD apathy [27]. While some RCT exercise-based interventions including Nordic walking showed an improvement in apathy scores [168,169,170], another RCT failed to demonstrate a significant improvement in apathy [171]. Repetitive transcranial magnetic stimulation (r-TMS) targeting either the supplementary motor area, the M1 area or the DLPFC has been found to improve apathy scores [10,172,173].

Altogether, the findings are inconclusive regarding the management of PD apathy. Larger RCTs taking into account the subtype of apathy are necessary.

## 8. Conclusions and Perspectives

Apathy is a multidimensional neuropsychiatric syndrome which may combine limbic and cognitive deficits that interact. It results from a complex interplay between PD profile, neurotransmitter dysfunction and therapeutic management [5,47]. Careful phenotyping of motor, cognitive and behavioral signs is an essential milestone in determining a patient’s clinical profile [155,156]. DA and serotonergic PET imaging and structural imaging may help to distinguish motivational dopa-responsive apathy from cognitive dopa-resistant apathy [15,16,153,174]. In the near future, the development of cholinergic and noradrenergic radioligands may facilitate better characterization of the neurophysiological substrate of cognitive apathy. Moreover, the use of high-density EEG may help to identify potential biomarkers of both limbic and cognitive apathy as well as compensatory mechanisms [175,176,177,178,179]. Finally, the search for the genetic variants involved in dopamine-dependent behaviors such as *OPRM1*, *DAT1*, *HTR2A* and *DDC*, or variants associated with a risk of cognitive decline such as *GBA* and *APOE*, could provide clinical–genetic models for predicting the risk of developing motivational or cognitive apathy [180,181,182,183,184,185].

## Data Availability

Not applicable.

## Abbreviations

ACC	Anterior Cingulate Cortex
AES	Apathy Evaluation Scale
AI	Apathy Inventory
AS	Apathy Scale
ASBPD	Ardouin Scale of Behaviors in Parkinson’s Disease
BG	Basal Ganglia
DA	Dopaminergic
DAT	Dopamine Transporters
DBS	Deep Brain Stimulation
DLPFC	Dorsolateral Prefrontal Cortex
D1Rs, D2Rs, D3Rs	Dopamine D1, D2, D3 Receptors
DRT	Dopamine Replacement Therapy
EBDM	Effort-Based Decision Making
GABA	Gamma-Aminobutyric Acid
GDBs	Goal-Directed Behaviors
GPe	Globus Pallidus Externus
GPi	Globus Pallidus Internus
ICD	Impulse Control Disorders
ISCTM	International Society for Central Nervous System Clinical Trials Methodology
LARS	Lille Apathy Rating Scale
MDS	Movement Disorder Society
MCL	Mesocorticolimbic
MPTP	1-methyl-4-phenyl-1,2,3,6-tetrahydropyridine
NAc	Nucleus Accumbens
NPI	Neuropsychiatric Inventory
OFC	Orbitofrontal Cortex
6-OHDA	6-hydroxydopamine
PD	Parkinson’s Disease
PET	Positron Emission Tomography
PFFs	Preformed α-synuclein Fibril(s)
PSP	Progressive Supranuclear Palsy
RCT	Randomized Controlled Trial
r-TMS	Repetitive Transcranial Magnetic Stimulation
SERT	Serotonin Transporters
SNc	Substantia Nigra Pars Compacta
SSRI	Selective Serotonin Reuptake Inhibitors
STN	Subthalamic Nucleus
UPDRS	Unified Parkinson’s Disease Rating Scale
VS	Ventral Striatum
vmPFC	Ventromedial Prefrontal Cortex
VTA	Ventral Tegmental Area
VTAc	Volume of Tissue Activated
